# Chromatin Alterations in Neurological Disorders and Strategies of (Epi)Genome Rescue

**DOI:** 10.3390/ph14080765

**Published:** 2021-08-04

**Authors:** Marcin Janowski, Małgorzata Milewska, Peyman Zare, Aleksandra Pękowska

**Affiliations:** Dioscuri Centre for Chromatin Biology and Epigenomics, Nencki Institute of Experimental Biology, Polish Academy of Sciences, Pasteur Street, 02-093 Warsaw, Poland; m.janowski@nencki.edu.pl (M.J.); ma.milewska@nencki.edu.pl (M.M.); p.zare@nencki.edu.pl (P.Z.)

**Keywords:** epigenetics, transcriptional regulation, cis-regulatory elements, nervous system, chromatin structure, histone modifications, CRISPR-Cas9

## Abstract

Neurological disorders (NDs) comprise a heterogeneous group of conditions that affect the function of the nervous system. Often incurable, NDs have profound and detrimental consequences on the affected individuals’ lives. NDs have complex etiologies but commonly feature altered gene expression and dysfunctions of the essential chromatin-modifying factors. Hence, compounds that target DNA and histone modification pathways, the so-called epidrugs, constitute promising tools to treat NDs. Yet, targeting the entire epigenome might reveal insufficient to modify a chosen gene expression or even unnecessary and detrimental to the patients’ health. New technologies hold a promise to expand the clinical toolkit in the fight against NDs. (Epi)genome engineering using designer nucleases, including CRISPR-Cas9 and TALENs, can potentially help restore the correct gene expression patterns by targeting a defined gene or pathway, both genetically and epigenetically, with minimal off-target activity. Here, we review the implication of epigenetic machinery in NDs. We outline syndromes caused by mutations in chromatin-modifying enzymes and discuss the functional consequences of mutations in regulatory DNA in NDs. We review the approaches that allow modifying the (epi)genome, including tools based on TALENs and CRISPR-Cas9 technologies, and we highlight how these new strategies could potentially change clinical practices in the treatment of NDs.

## 1. Epigenetic Mechanisms Driving Cell Identity and Disease

In a seminal paper from 1942, Conrad Hal Waddington suggested the term “epigenetics” to describe the ensemble of outcomes that result from the functional interactions between genes which produce a mature organism during development [1]. At that time, the mechanisms underlying epigenetics remaindered largely obscure [2]. Evidence accumulated ever since indicates that factors orchestrating chromatin activity, and thereby gene expression, are vital players making up the epigenome allowing to establish and maintain cell identity during development and adult life [3,4]. The more recent definition that we will consider throughout this manuscript describes epigenetic events as the *structural adaptation of chromosomal regions so as to register, signal and perpetuate altered activity states* [5].

In eukaryotes, DNA associates with a core octamer of histones to form a nucleosome and the array of nucleosomes (the 10nm fiber) constitutes the basic unit of chromatin organization in the interphase [6,7,8,9,10,11,12]. This structural arrangement of DNA has profound consequences from the transcriptional standpoint [13,14,15,16] and the modifications of nucleosomes play a pivotal role in the regulation of gene activity [17]. Multiple layers of regulation of chromatin structure have evolved to ensure that only the proper subset of genes is switched on in a particular cell type [18]. At the most basic level, transcriptional control relies on the binding of sequence specific transcription factors (TFs). Since only a fraction of TFs can bind nucleosomal DNA [19], TF binding frequently requires removal of nucleosomes occluding the TF’s target sequence. TFs recruit transcriptional co-activators or co-repressors [20] which modify the local chromatin structure, thereby allowing for control of the on and off states of genes. Eukaryotic cells are endowed with additional mechanisms, which act seemingly without the direct implication of TFs but involve factors that modify DNA and chromatin proteins, allowing for the induction and the maintenance of stable gene repression, these strategies include for instance position effect (see below [21,22,23,24]) and can operate across multiple generations [25]. Modifications of the epigenetic machinery often result in aberrant functions of the central nervous system (CNS) and contribute to numerous neurological disorders (NDs).

We outline the implication of chromatin modifications in transcriptional regulation. We focus on the implication of the epigenetic mechanisms in NDs. We review the therapeutic approaches that take advantage of specific changes in the epigenome of NDs and discuss the most recent data uncovering the possible mechanistic implication of the genetic risk variants in NDs. Finally, we highlight promising cutting-edge technologies, directed at (epi)genome engineering, which can potentially be harnessed for the design of the future therapeutic strategies to treat NDs.

### 1.1. Mammalian DNA Cis-Regulatory Elements Classification and Chromatin Signature

**Figure 1:**
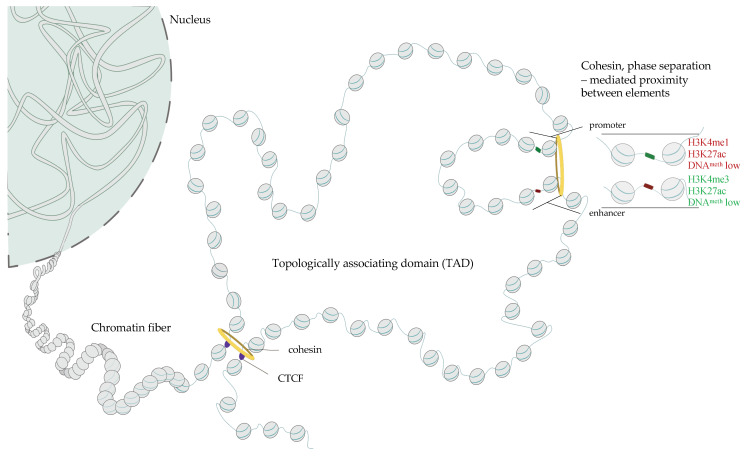
Chromatin 3D structure in the cell in relation to transcriptional regulation. Promoter-enhancer loops form within the context of the topologically associating domains (TADs), which arise as a consequence of the action of the cohesin complex. The binding of CTCF blocks the displacement of the cohesin ring thereby defining the location of the TADs boundaries. Active promoters and enhancers are devoid of DNA methylation. The two classes of elements display a specific histone modification signature featuring high levels of H3K27ac (both enhancer and promoters) and unique pattern of H3K4me3 modification (promoters being highly enriched and enhancers displaying non-detectable or low level of this modification). H3K4me1 is particularly enriched at active and poised enhancers. When active, promoters and enhancers can often display an increased spatial proximity in the cell nucleus.

TFs can act as activators or repressors, and it is the expression of unique combinations of TFs in each cell type that allows for eliciting and maintaining a defined cell identity [26]. TF binding sites (TFBS) are frequently clustered in the genome and the sequences enriched in TFBS often act as cis-regulatory elements (CREs). CREs can activate gene expression (enhancers and promoters) or silence it (silencers and, indirectly, insulators). Moreover, CREs can be in various positions with respect to their cognate transcription start site (TSS), including in 3’ and 5’ end of the regulated gene, intron of either the target or of an unrelated gene. Enhancers, silencers and insulators are frequently distal (up to megabases (Mb) apart from their target gene); they can be found in either 3’ or 5’ direction with respect to the 5′ end of the promoter they regulate [27] while promoters flank the gene’s TSS. Inactive or poised promoters are enriched in methylated DNA [28,29,30] and heterochromatic histone marks including H3K9me3, H3K27me3 [31] and feature overall histone deacetylation [32,33]. In contrast, active regulatory elements display decreased DNA methylation [34], increased accessibility to nuclease digestion [35,36,37] and enrichment in specific histone modifications including H3K27ac, H3K4me3 and H3K4me1 and binding of TFs rendering these regions more sensitive to nuclease action (Figure 1) [31,38,39,40,41,42,43]. Taken together, genome-wide mapping of chromatin openness and enrichment of H3K4me1, me3 and H3K27ac and CTCF allows for identifying promoters, enhancers and insulators in an unbiased manner.

### 1.2. DNA Methylation and Hydroxy-Methylation

In mammals, the carbon 5 in the cytosine’s aromatic ring is the major target of methylation (5mCy) [44], even though adenine can also undergo this modification [45]. DNA methylation is primarily deposited at cytosines that precede guanines (CpGs); CpG dinucleotides frequently occur in stretches—CpG islands often located close to gene promoters [29,46,47]. Secluded CpGs are largely methylated across the genome, while the ones within the context of CpG islands are most frequently unmethylated. DNA methylation is implicated in stable gene silencing and is important in the context of X-chromosome inactivation [48,49,50], allele-specific gene expression (imprinting) [51] and transcriptional regulation during development [29,30,48,52]. The reaction of DNA methylation is catalyzed by DNA methyltransferases [53,54] and occurs either *de novo* [53,55,56,57,58] or during genome replication [54]. 5mCy is recognized by proteins containing the methyl binding domains (MBDs) and the protein MeCP2. 

The removal of methyl groups from DNA correlates with transcriptional onset. DNA methylation can be lost passively, during cell division or actively via enzymatic oxidization of carbon 5 within the aromatic ring of 5mCy, a reaction catalyzed by ten-eleven translocation (TET) proteins 1–3 [59]. The resultant 5-hydroxymethyl cytosine (5hmCy) is stable in the cell, non-mutagenic and particularly abundant in the brain [60]. Further oxidation of 5hmCy by TET proteins results in the formation of 5-formylcytosine (5fmCy) and next 5-carboxycytosine (5cmCy) [61,62], which are recognized by dedicated factors [63,64] and rapidly processed in the cell [61,62]. Both 5fmCy and 5cmCy are then excised by thymine DNA glycosylase (TDG)-mediated base excision repair (BER) mechanism [65].

### 1.3. Histone Modifications

Histone proteins can undergo a plethora of post-translational modifications that are deposited or removed by specialized multicomponent complexes. Histone acetylation and methylation at the unique lysine residues are related to transcriptional regulation (Figure 1) and the presence of a set of modifications at promoters and enhancers is related to their activity (see below). Other histone modifications including phosphorylation, ubiquitination, crotonylation, and sumoylation play diverse roles in chromatin biology [66,67] and will not be reviewed in the context of this manuscript. 

Histone methylation is recognized by the royal family domains (including MBT, PWWP, Tudor and chromodomain), plant homeodomain (PhD) finger domains and zinc-finder and WD repeat modules. Acetyl-lysins are recognized by bromo- and YEATS-domains. Chromatin regulatory complexes are typically composed of multiple proteins with domains recognizing modified histones, thereby acting as nodes ensuring crosstalk between epigenetic pathways. Likewise, histone modifications alter three-dimensional (3D) chromatin arrangement [12,16,68,69] and, at a smaller scale, the structure of the nucleosome particle thereby impacting gene activity [67].

#### 1.3.1. Histone Acetylation

Since the discovery of acetylated histones in vivo, it was postulated that this modification is implicated in the regulation of chromatin structure and activity [70]. The discoveries that histone acetyltransferases (HATs) constitute essential transcriptional co-activators [71,72,73] and that histone deacetylases are frequently implicated in the activity of co-repressor complexes [74,75,76,77,78,79,80] have further cemented this link. The acetylation of H3 and H4 is overwhelmingly associated with gene activation, genome-wide analyses of the distribution of acetylated histones revealed localized pattern of enrichment at active regulatory elements including enhancers [31,40,41,81], binding of p300 and CBP histone acetyltransferases is in fact amongst the most reliable markers of tissue-specific enhancer activity [82,83,84]. Histone acetylation is reversed by histone deacetylases (HDACs). As we will discuss below, several epigenetic drugs target HDACs to inhibit their activity, thereby restoring gene expression. 

#### 1.3.2. Histone Methylation

The transfer of methyl groups from S-adenosyl-methionine to lysins and arginines in histones is catalyzed by the SET domain in histone lysine methyltransferases (KMTs) including the trithorax-like proteins such as KMT2A and histone arginine methyltransferases (PRMTs). These enzymes deposit up to three methyl moieties on lysins and up to two methyl groups on arginine. Histone methylation is linked with both gene activation and silencing. For instance, histone 3 lysine 4 tri-methylation (H3K4me3) is enriched at promoters of active genes while H3K36me3 marks gene bodies of actively transcribed genes [31,85,86]. On the contrary, H3K27me3, deposited by the HMT EZH2 within that Polycomb Repressive Complex 2 (PRC2) is enriched at promoters of silenced loci. H3K9me2 and me3 are deposited by HMT *SUVH1*. They are implicated in the silencing of the repetitive sequences and are present at silenced loci within facultative heterochomatin. 

Methylation competes with acetylation for lysine residues in histones and the replacement of one kind of modification by another can lead to a fundamentally different functional outcome. For instance, methylation of lysine 27 in histone 3 (H3K27me3) silences enhancers while the acetylation of the same residue is linked with their activation [40]. 

### 1.4. Nucleosome Positioning

The formation of nucleosomes can inhibit the binding of TFs and consequently transcription from a promoter [13,14,15]. Several multiprotein complexes are present in the cells and act to displace or evict nucleosomes in an ATP-dependent manner [87]. Based on the composition, chromatin remodelers are divided into four major families: SWI/SNF (switch/sucrose non-fermentable, also called BAF complexes) [88,89,90,91], ISWI (imitation SWI [92,93,94,95,96,97]), INO80 (SWI2/SNF2-related (SWR)), and CHD (chromodomain helicase DNA-binding). What unites these families of complexes is the presence of ATP-dependent DNA helicase from the SNF2 family. All these complexes display tissue specific composition. There are also several orphan families of chromatin remodelers which include the ATRX factor. Chromatin remodeling complexes are critical for proper activity of promoters [98,99], enhancers [100,101,102,103,104,105,106,107,108], and insulators [109,110,111,112]. Their action is related to both repression of CREs [113,114] and cell type specific gene activation. Chromatin remodelers are frequent target of mutations in cancer [115] and the deficiencies in their activity are related to multiple NDs (Table 1). 

### 1.5. Three-Dimensional Structure of Chromatin–The Role of CTCF, Cohesins and Phase Separation in the Buildup of Genome Topology 

Cognate pairs of CREs can be located at considerable genomic distances and a substantial fraction of enhancer-promoter pairs are separated by more than 10kb of DNA [220,221]. For instance, limb specific enhancer of the sonic hedgehog gene (*SHH*), an important morphogen implicated in the development of the nervous system, is located 850 kb away from the *SHH* promoter [222]. The current model posits that the functional interaction between CREs relies on a physical contact between these elements and one of the open questions in the field is how the cognate CREs are tethered to each other to maintain gene expression patterns. The factors that control associations between CREs in the 3D space are most likely of central importance in health and disease. 

Over the past decade, there has been a considerable progress in the understanding of the mechanisms that guide the formation of contacts between genomic regions in the 3D space of the cell nucleus [223]. High resolution, genome wide chromatin conformation capture experiments including Hi-C [224], revealed that at the genomic distances of up to several megabases, genomes fold into domains of high self-contact which were deemed topologically associating domains (TADs [225,226,227], Figure 1). The fact that the cognate CREs frequently reside within the same TAD [34,228,229,230], led to a supposition that these structures constitute functional units of genome organization. Zooming out to larger genomic length-scales, Hi-C revealed that chromosomes are organized into two major compartments the euchromatic compartment A and a more heterochromatic compartment B [224,231], which can be further subdivided into sub-compartments [232]

Nuclear topology arises as a consequence of the interplay between the formation of compartments that bridge regions of similar activity to each other, and the cohesin-mediated loop extrusion [233,234,235,236,237] responsible for the formation of TADs [238,239,240,241,242] in an energy-dependent fashion [243,244,245,246] (Figure 1). These two activities counteract each other [238,239,240,241,242] and the compartmental interactions are disrupted by the loop extrusion machinery. Cohesins bind to DNA and their movement produces a growing chromatin loop (Figure 1). The process stops when cohesin complex encounters a CTCF protein bound to DNA motif that faces them (Figure 1). A stable loop assembles when both loop anchors are occupied by CTCF and the motifs of these two binding sites face each other [247].

Loop extrusion dismantles homotypic interactions that underlie compartments [238,239,240,241] but how compartments assemble remains unclear. Recently, the process phase demixing or liquid phase–liquid phase separation (LLPS) has been implicated in the regulation of nuclear activity and chromatin topology. LLPS occurs when two liquids characterized by distinct physicochemical properties separate. LLPS results in the emergence of droplets or condensates where high concentrations of the separated molecules can be attained [248,249]. LLPS contributes to genome arrangement in various organisms across phyla [250]. Nucleoli [251] and heterochromatin foci [252] for instance form as a consequence of LLPS. Numerous factors that regulate enhancer activity and enhancer-promoter dialogue including mediator complex, TFs, mRNA polymerase harbor intrinsically disordered regions (IDRs) and form condensates in the nucleus [253,254,255,256,257,258]. A disruption of the LLPS affects promoter activity [253,255]. LLPS emerges as an essential property of chromatin-modifying proteins and transcriptional machinery.

## 2. Genome Activity in Neurological Disorders

Neurological disorders (NDs) constitute a diverse group of conditions that affect the functions of the central and peripheral nervous systems. Depending on the condition, NDs can manifest themselves with mental retardation, intellectual disability, seizures, and as in the case of neurodegenerative diseases (NDGDs) a gradual and progressive decline in cognitive or motor functions. The most widespread and well described NDGDs include Alzheimer’s disease (AD), Parkinson’s disease (PD), Huntington’s disease (HD) as well as amyotrophic lateral sclerosis (ALS), spinal muscular atrophy (SMA), and Friedrich’s ataxia (FRDA). Neuropsychiatric diseases (NPDs) feature alterations in brain physiology and functions. NPDs including schizophrenia (SZ), bipolar disorder (BD), major depressive disorder (MDD), attention deficit hyperactivity disorder (ADHD), specifically affect emotions, and in the context of SZ the feeling and the definition of the self.

NDs can be related to dysfunction of a single gene (Table 1). However, frequently, the genetic underpinnings of NDs are more complex. For instance, there is a strong hereditary component to NPDs [259,260] and many variants have been associated with an increased risk of developing these diseases. However, the mechanisms implying the genetic variants linked to NPDs are frequently multilayered and complex and hence not fully understood. Likewise, the fact that the genetic alterations linked with NPDs are typically of low prevalence amongst patients [113,261,262,263] constitutes a major challenge for the classification and efficient treatment of these conditions.

### 2.1. NDs Frequently Feature Defective Machinery Establishing and Reading Histone and DNA Modifications

Mis-regulation of gene expression likely constitutes a pivotal event leading to the onset of NDs and their exacerbation [264]. Mutations in chromatin-related factors are significantly over-represented in disorders featuring altered brain physiology and functions [183,262,265,266,267,268,269,270] (Table 1 and references therein). At the same token, genes mutated in NDs, frequently encode factors implicated in the normal development of the nervous system [271,272,273]. Therefore, the understanding of how the chromatin-related factors, which are also linked to NDs, contribute to the normal development of the brain will likely help refine clinical strategies to diagnose and treat these conditions (Table 2) [271,274]. For instance, Rett syndrome, one of the most common causes of mental retardation in women, is caused by a mutation in the X-linked *MeCP2*, encoding a transcriptional regulator that binds to methylated DNA, and together with HDACs establishes repressive chromatin environment at CpG methylated promoters. Nicolaides–Baraitser and Coffin–Siris syndromes are caused by mutations in *SMARCA2* and *ARID1B* respectively, the components of the SWI/SNF chromatin remodeling complex which orchestrates gene expression (Table 1).

Tightly established protein level of chromatin-related factors is a prerequisite to the normal functioning of the CNS. Erroneous protein dosage of even the same chromatin regulator can lead to distinct phenotypic manifestations. For instance, loss of one of the copies of the genes encoding in CBP or P300 histone acetyltransferases is the underlying cause of the Rubinstein–Taybi syndrome while the duplications in the region encoding CBP lead to a characteristic 16p13.3 syndrome [275,276] featuring unique characteristics including mild mental retardation. Likewise, multiple NDs are not directly caused by mutations in the coding sequence of chromatin factors, but instead feature their transcriptional mis-regulation and distorted nuclear activity. For instance, HDAC1, which catalyzes the removal of acetylation from histone proteins, is significantly overexpressed in the brain of SZ patients [277,278,279]. PD, a late-onset progressive neurodegenerative disorder caused by mutations in α-synuclein which leads to its sequestration and the assembly of the Lewy bodies [280]. Lewy bodies are toxic to neurons, causing their decline. Consequently, PD is characterized by a loss of coordination and decay of motor skills. The formation of Lewy bodies also correlates with a sequestration of DNMT1 to the cytoplasm, subsequent decrease in nuclear DNMT1 and global hypomethylation of the genome in neurons [281]. More recently, genome-wide histone acetylation analysis revealed a general promoter hyperacetylation in the prefrontal cortex of patients suffering from PD [282]. HD is another example of a late-onset progressive NDGD. HD is caused by an expanded polyglutamine repeat sequence in the huntingtin protein (HTT). The mutant HTT, unlike its wild-type counterpart, enters the nucleus where it binds and inhibits HATs, leading to a decrease in H3 and H4 histone acetylation, and thus to a global transcriptional mis-regulation including the silencing of neuronal genes [283,284]. HD features changes in DNA methylation; in comparison to wild type cells, polyglutamine-expanded HTT (STHdhQ111/Q111) cell line displays an increased DNA methylation at more that 21 thousand sites including promoters of essential to neuron differentiation, neurogenesis and transcription [285]. DNA demetylation in this HD model is much less pronounced and affects around 10,000 sites [285].

AD is one of the most common NDs and features a progressive impairment of cognitive functions. The formation of amyloid plaques that contain beta-amyloid protein is one of the hallmarks of AD and is mediated by the enzymatic cleavage of the amyloid precursor protein (APP) by β-secretase (BACE1). The overexpression of APP might be caused by a hypomethylation of its promoter’s DNA [286]. APP can also be elevated due to a less direct, epigenetic effect. For instance, promoter hypomethylation of transmembrane Protein 59 (TMEM59) in AD leads to its upregulation. As a consequence, APP cleavage by α- and β-secretase is inhibited, and APP deposition enhanced [287]. Remarkably, AD features a global activation of chromatin. Multi-omics analysis of AD and healthy brain samples revealed upregulation of chromatin factors including HATs (CBP and p300) and TRRAP (SAGA–ATAC complex subunit), HDACs (SIRT1 and HDAC4), histone methyltransferases (CXXC32) and histone demethylases (JMJD6) and gain in H3K27ac and H3K9ac at the promoter of *CBP* and *CTCF* [288,289,290,291]. Likewise, fine mapping of regulatory element chromatin landscape in individuals at different stages of AD revealed frequent DNA hypomethylation at enhancer elements linked with AD and potential mechanisms driving AD including the upregulation of β-secretase though transcriptional gain of *DSCAML1* [292]. Altogether, deregulation of gene expression and chromatin signature are most likely essential for the development and progression of NDs.

### 2.2. Chromatin Topology Is Related to Neuronal Function and Can Be Affected in NDs

Mutations in CTCF and in the components of the loop extrusion machinery including cohesins and auxiliary factors that regulate the loading and the stability of the cohesin complex on chromatin, manifest themselves with multiple congenital defects including but not limited to mental retardation (Table 1). Likewise, as we saw, genetic alterations of factors impacting promoter-enhancer dialogue including mediator and histone acetyltransferase P300 result in neurological syndromes (Table 1). To harness the potential of these observations in clinical practice, we need to deepen our understanding of the interplay between the topology of the genome and transcriptional regulation. One important aspect of this question is to define how chromatin arrangement contributes to the action of enhancers and promoters and how insulators impact these transactions [223,293]. One of the ways to impact the 3D distance between genomic regions is to establish an extrusion-mediated CTCF-CTCF loop. Indeed, CTCF-mediated loops have been related to the control of promoter-enhancer contacts in various contexts [243,294,295,296,297,298] including the control of protocadherin gene expression during neural development [299]. Furthermore, CTCF binds to numerous genes underlying neuronal functions; CTCF knockdown in the hippocampus leads to transcriptional mis-regulation of over 700 loci correlating with an impairment in learning and memory in the mouse model [300]. Whether CTCF acts to impact promoter-enhancer loops at these loci remains open. In murine models, neuronal activity elicits the formation of promoter-enhancer loops at a subset of regulated loci [301,302], and leads to multiple changes in compartmental interactions. Genetic ablation of cohesin affects this process and leads to a reduction of promoter enhancer loops [301] and the removal of cohesins correlates with impaired motor learning [301]. These observations indicate that loop-extrusion-mediated chromatin topology plays an active role in the regulation of higher-level brain functions.

CTCF is the only known mammalian insulator to date and the insulatory functions of CTCF rely on the presence of cohesins [303,304]. Consistent with a convergent role of CTCF and cohesins in the formation of TADs, a disruption of either of the two mechanisms leads to loss of TAD insulation [238,239,240,305,306,307]. CTCF loss can cause a misalignment of transcriptional enhancers and an aberrant activation of nearby genes [294,308]. Alteration of CTCF binding can be caused by the mutation in the CTCF gene, changes in the level of CTCF expression level or by the modification of its binding site architecture. The latter can intervene at loci implicated in NDs. Modeling of the neuronal and astrocytic lineage function using induced pluripotent stem-cell models revealed an overrepresentation of genetic variants related to NPDs at the anchors of loops assembled specifically in iPS-derived neurons suggesting an important role of chromatin architecture in neuronal functions [309]. Whether the same phenomenon occurs in the brain of diseased individuals remains to be assessed but recent observations strongly link CTCF function to brain development and NDs [310]. At the 7p21 locus associated with neurodegenerative disease frontotemporal lobar degeneration, a mutation that precludes the binding of CTCF results in an altered chromatin topology featuring an increase in the regulatory interactions of the promoter of *TMEM106B* likely leading to an enhanced expression of *TMEM106B* and cytotoxicity [311]. The fact that disease risk variants related to NDGNs are enriched with CTCF binding events [311] leads to an assumption that there will be many more examples of loci that are transcriptionally mis-regulated as a consequence of aberrant recruitment of CTCF.

### 2.3. Next Generation Brain Regulome Maps to Link Genetic Variants to Essential Loci and Identify New Genes Underlying ND

There are several strategies that allow to uncover genes and CREs that are likely to contribute to NDs. Genetic linkage mapping can give hints as to the mechanisms of the NDs, help early diagnosis, and possibly reveal the most suitable intervention points for clinical practice. Genome wide association studies (GWAS) allow the discovery of common genetic variants, including single nucleotide polymorphisms, insertions, deletions, or duplications, which underlie traits, including disease susceptibility. GWAS relies on sequencing of the genome of the diseased and healthy individuals and a subsequent computational identification of the regions in the genome that segregate with a trait or a disease. Remarkably, while numerous GWAS hits affect protein-coding regions [312], the majority of disease risk variants, including loci linked with NDs, are often non-coding and frequently feature regulatory element chromatin signature and activity [313,314,315,316,317,318,319] further highlighting the contribution of transcriptional regulatory mechanisms to the etiology of NDs. Regulatory mechanisms that drive disease-related changes in gene expression can in turn be identified through the analysis of the expression quantitative trait loci (eQTLs) in which the transcriptomes of healthy individuals and patients are compared in the function of the genetic changes in the intergenic CREs. 

Having established the cartography of CREs affected in NDs, the next step is to functionally link these elements to genes and pathways that can be targeted clinically. This task is challenging and addressing it constitutes one of the most outstanding goals in the field [320]. Several international consortia, including the *Psychencode* (https://www.nimhgenetics.org/resources/psychencode, accessed on 23 July 2021), *ENCODE* and *GTEx* [321] have been established to determine the implication of the regulatory element mutations and transcriptional deregulation in NDs. Multiple exciting examples of how functional genomics, by combining chromatin activity and architecture mapping, can help unravel the mechanisms of action of genetic hits related to chosen NDs highlight the great power of multiomics to address ND and suggest possible clinical intervention points. 

#### 2.3.1. Comparison of Wild Type and ND Chromatin Can Help Identifying Essential Pathways Related to NDs

Essential genes and regulatory elements underlying NDs can be identified via a direct comparison of healthy and disease brain tissue. For instance, AD-specific H3K27ac peaks frequently overlap AD GWAS hits with prominent examples, including putative enhancers mapping in the vicinity of *MAPT*, *PSEN2*, genes controlling amyloid-beta and tau biology [289], as well as genes related to synaptic transmission and immune response [322]. Likewise, single nucleotide polymorphisms associated with PD overlap enhancer elements at the α-synuclein (*SNCA*) and *PARK16* genes, both which are implicated in the neuropathology of PD [323]. Genetic replacement of a wild-type *SNCA* enhancer, by its version displaying a significant association with PD, results in an increased level of *SNCA* expression in neurons derived from the genetically modified iPS cells [324]. Sequence analysis of the risk variant suggests that the up-regulation of *SNCA* in PD might arise as a consequence of a loss of binding of the EMX2 and NKX6-1 TFs that repress *SNCA* expression in neurons [324]. 

Due to its heterogeneity, SZ is an extremely challenging and not well understood ND; hence, there is an urgent need to determine which genes are essential to this syndrome. Like other NDs, SZ features significant changes in gene expression [325], which in part could be explained by an aberrant activation of key enhancers [326,327]. For instance, a risk allele within the first exon of the *NDUFA6* gene, disrupts the production of the NDUFA6 protein by inducting nonsense-mediated decay of its messenger RNA. Remarkably, apart from acting on the NDUF6 transcript, this mutation also hampers the binding of YY1 transcriptional regulator, which in turn has leads to a reduced expression of *NAGA*, a gene located 32 kb upstream of the enhancer [328]. Interestingly, *NAGA* itself is a SZ risk gene, uncovered through eQTL analysis. Hence, the analysis of 3D chromatin arrangement helped identifying the possible mechanism that drives *NAGA* deregulation in SZ [328]. Altogether, the comparison of healthy and diseased tissue can illuminate on the mechanisms driving NDs. However, this approach cannot unveil the longitudinal effects of generic mutations that intervene at a restrained time window during brain development. Hence, it will miss relevant molecular events and markers for diagnostic purposes allowing to manage the disease at its earliest stages. One can address this question by functional analysis of the impact of GWAS on brain biology.

#### 2.3.2. From GWAS to the Causal Gene

Having only the information about healthy tissue, how do we navigate the ocean of GWAS to identify the most interesting targets for follow up studies and clinical practice? There are several approaches that we can harness to accelerate the discovery in this domain. These include computational approaches that allow to prioritize variants for functional follow-up studies based on Bayesian fine-mapping [329] and regression analysis [330]. Likewise, summary association statistics inferred through GWAS and transcriptomic effects from eQTL analysis can be considered jointly, which enhances the power to detect interesting candidate loci [331,332]. Another approach calls to prioritize GWAS hits by assessing their impact on TFBS, thereby greatly facilitating the identification of most likely candidates that orchestrate transcriptional mis regulation in ND [333]. Comparative sequence analysis can also reveal fundamental mechanisms underlying NDs. Exemplifying this, a relatively recent global analysis of risk variants of SZ that specifically affect TFBS, revealed 132 loci whereby the sequence motif recognized by a TF is disrupted by the disease-linked mutation. Interestingly, a substantial fraction of these sequence changes affects the binding sites of CTCF and cohesins [326] strongly suggesting an essential role of chromatin topology in SZ. The classification and the identification of the most promising variants of CRE can be substantially aided experimentally. The impact of a mutation in a CRE can be then tested by assessing how it affects the binding of a TF in for instance Electromobility Shift Assay (EMSA). The impact of the genetic mutation on the CRE activity can be tested directly by reporter assays whereby the CREs are tested for their capacity to regulate the expression of a reporter gene (luciferase or a fluorescent protein) controlled by a weak promoter. Reporter assays can be used to test individual sequences or thousands of sites simultaneously like for instance in the massively parallel reporter assays (MPRA) [334,335,336]. 

Enhancers are typically at vast genomic distances from promoters and do not necessarily regulate their closest gene. When active, enhancer-promoter pairs can display an increased spatial proximity and the chromatin conformation assays can help to link them to each other [34,220,337,338,339,340,341]. Using PLAC-seq, which allows to identify the interactions between genomic sequences at high regulation, Nott et al., uncovered the most likely enhancer-promoter pairs in several major cell types in the human brain [342]. One of the essential conclusions of this study, somewhat in agreement with expectations [220,343], is that enhancers frequently do not simply act upon the closest gene. For instance, an intronic GWAS AD risk variant within the gene *SLC24A4* that overlaps a putative enhancer tends to interact with several genes located >200 kb in the 5′ direction (*CPSF2*, *ATXN3* and *TRIP11*). Strikingly, it does not form contacts with the promoter of *SLC24A4* which was assigned to it by GWAS analysis. 

The analysis of the 3D conformation of chromatin in developing neurons can highlight new functions of regulatory elements linked to NDs at the early stages of brain development. In SZ, chromatin conformation capture connected regulatory elements mutated in patients to new genes including acetylcholine receptors. This analysis revealed the association of SZ risk loci with essential neuronal TFs such as *FOXG1* intervening in the cortical development [343]. Similarly, Golgi phosphoprotein 3-like (*GOLPH3L*), gene implicated in trafficking of key neuronal signaling receptors to the cell membrane has recently been linked with SZ through the discovery of a mutation in its putative enhancer in SZ patients [344]. The possible implication of *GOLPH3L* in SZ would have otherwise been overlooked, illustrating once more the power of epigenomics to uncover new factors related to NDs. The integrative analysis of chromatin activity and gene expression during the normal brain development is enhancing our mechanistic understanding of numerous other NPDs including bipolar disorder [345] and autism [346], further strengthening the association between aberrant transcriptional control during brain development and NPDs. 

#### 2.3.3. Towards High Resolution Functional Genomics of NDs 

Multiple cell types, including but not limited to neurons, micro and macroglia (including astrocytes and oligodendrocytes) make up the CNS. Yet, the precise number and the molecular identity of the distinct cell populations in the human brain is not clear. To understand the implication of the risk variants in NDs, it will be essential to disentangle to impact of the disease-related genes and cis-acting elements in high resolution with respect to the cell types present in the CNS. Towards addressing this need, Sullivan et al., have performed in-vitro experiments and assessed how genes linked to AD affect the production of the extracellular β-amyloid by neurons and astrocytes [347]. The authors uncovered astrocyte- and neuron-specific role of the tested genes in regulating the levels of β-amyloid highlighting unique contribution of these cell lines to AD [347]. In a genome-wide study, Nott et al., have addressed the possible mechanistic implications of distinct cell types in NDGDs. Based on surface marker expression, the authors separated brain cortical cells into the major lineages including neurons, astrocytes, oligodendrocytes and microglia. Then, using ATAC-seq to identify open chromatin regions and ChIP-seq to assess the profiles of H3K4me3 and H3K27ac, Nott et al., mapped active enhancers and promoters in these lineages. Remarkably, GWAS loci related to NPDs preferentially overlapped enhancers active specifically in neuronal cells, while GWAS hits related to AD were primarily intersecting regulatory elements active in microglia [342] further cementing the link between microglial disfunction and AD. 

Brain is highly regionalized and cells from its different parts display unique transcriptional programs which likely leads to unequal susceptibility to succumb to the functional impact of the genetic alterations causing ND. Therefore, to understand the genetics of ND, we need not only cell-type, but also region-specific regulome maps. Creyghton and colleagues have provided enhancer maps of 136 regions of the adult human brain [323]. Brain regions cluster according to putative enhancer activity and the segregation corresponds grossly to anatomical structures. While most of the enhancers are shared between regions, a substantial number of interesting CREs is region-specific. Focusing on enhancers overlapping GWAS hits related to PD, the authors uncovered new α-synuclein enhancer, active in the majority of the sampled brain regions, which overlaps a genetic variant related to PD. This element also interacts with *GPRIN3* a subunit of the NMDA glutamate receptor which has been considered as a potential target in PD treatment [323]. Interestingly, the activity pattern of the putative enhancer of the *PARK16* gene, the most significant PD-associated polymorphism within CREs uncovered in this study, was more restricted to the cerebellum. Through chromosome conformation capture in cerebellum, the enhancer at *PARK16* was further connected to other genes including *RAB7L1* and *MK2* which are both related to neurodegeneration [323]. Hence, detailed regulome maps of enhancer-promoter dialogue will likely help us to understand the implication of disease linked loci in NDs and the intersection of data obtained from different angles constitutes the most powerful tool to determine the genetic variants that are the most susceptible to underlie a trait including the development of NDs [348].

Anticipating the existence of a substantially larger spectrum of cellular states and identities in the CNS that are in fact known to date, can we go even further and propose an unbiased classification of risk loci to cell types? New technologies including single cell/nuclei RNA-sequencing, single cell assay for activity by transposase coupled with sequencing (ATAC-seq) [349] or spatial transcriptomics allow to assess the transcriptomic and epigenomic signatures of the individual cells [350,351], revealing the molecular identity of cell types in the CNS [352,353,354,355,356,357], and the spatial architecture of the CNS tissues [358,359]. Single cell approaches can highlight alterations of cellular function in diseased conditions [360,361,362,363,364,365] and reveal the importance of otherwise overlooked cellular fates in NDs [366] in part due to their superior power to detect differences in gene expression as compared to bulk approaches [360]. In the future, single-cell approaches allowing to map spatial conformation of the genome will contribute to further clarify the dialogue between enhancers and promoters in challenging cell populations in the brain [367,368]. Moreover, the joint information about the genotype and expression in single cell/nuclei RNA-sequencing experiments will help identify genes impacted by a disease-linked genetic variant [353,369] and hopefully will lead to a better understating of the regulatory landscape of genes that remain difficult to assess using the current technologies and require large scale sequencing approaches [329]. The far-reaching potential of such analyses to functionally annotate GWAS hits will likely allow us to paint a comprehensive picture of the genetic underpinnings of NDs. Combined with the unique capabilities of new technologies of genome and epigenome engineering, this knowledge will be instrumental to design new targets for clinical intervention strategies [320].

### 2.4. Targetting Global Epigenetic Signature in NDs

Drugs that target various epigenetic pathways (epidrugs) can display beneficial impact on brain functions in the context of several NDs (Table 2). The inhibitors of HDACs (HDACi), including sodium butyrate (SB, which blocks HDAC1 and 2), MS-275 (which blocks HDAC1 and 3), suberoylanilide hydroxamic acid (SAHA, Vorinostat, non-specific HDACi) and valproate acid (VA, which inhibits many pathways, including HDACs) can readily cross the blood brain barrier (BBB) and display beneficial impact on the CNS [370]. Despite a global character of their action, epidrugs can have a beneficial impact in the animal models of NDs and perhaps alleviate the syndromes in ND patients. Multiple clinical trials have been initiated to date aiming to determine the usefulness of epidrugs in ND treatments (Table 2).

Numerous examples support the favorable impact of epidrugs on ND progression. For instance, experiments performed on a transgenic mouse model of AD, showed that in comparison to the vehicle-treated animals, SB can help restore learning and memory, decrease tau protein phosphorylation, increase expression of associative learning-associated genes and restore histone acetylation in the hippocampus [371,372,373]. Likewise, SB and MS-275 can alleviate depressive behavior in AD and act to increase sucrose preference following repeated stress. The beneficial effect of HDACi is, at least in part, mediated by an enhancement of the acetylation of histone 4 at the promotor region of Transthyretin (*Ttr*) and a subsequent increase in *Ttr* leading to a disruption of beta-amyloid plaques [399,400].

In the mouse model of HD, treatment with SAHA or VA leads to an improved survival and motor performance if the treated mice compared to the control littermates [384,401]. In HD model mice (N171-82Q) phenylbutyrate treatment reduces neuronal atrophy, increases histone 3 and 4 acetylation and mice survival [374]. Likewise, sodium butyrate treatment can alleviate PD symptoms in the mouse and rat models and improve in cognitive deficits in pre-motor stage of the disease [402].

Depression has been linked with changes in DNA methylation [403]. The inhibition of DNA methylation by 5-aza-2-deoxycytidine leads to improvement of disease symptoms but the precise mode of action is unclear. Brain-derived neurotrophic factor (BDNF) is one of the essential growth factors that orchestrate the functions and viability of neurons. The antidepressant effect in global DNA-demethylation in behavioral tests might be due to an increase in the expression of BDNF in the hippocampus upon loss of methylation [396,404]. Altogether, epidrugs constitute a potentially beneficial strategy to treat NDs. However, their relatively unspecific character together with the fact that epidrugs impact the expression of only a fraction of genes, calls for more tailored approaches allowing to impact the expression of precisely chosen sets of genes in defined cell types.

## 3. Precision Medicine for (Epi)genome Engineering and Their Possible Application to Treat NDs

Development of new compounds that target epigenetic modifiers more specifically will lead to better defined clinical strategies. However, the genetic data indicates that NDs constitute a broad spectrum of syndromes featuring highly patient-specific genetic alterations. Therefore, it will be challenging to target such a vast repertoire of diseases with a single compound. One way out is to define the essential genes that are deregulated in a disease and act specifically at these loci to restore their expression level. 

Specificity of the regulation of gene expression in the cell relies on the recognition of a unique DNA sequence by a transcription factor. Hence, one way to modify the activity of a particular locus, for instance a regulatory element inactivated by a mutation, would be to engineer a DNA-binding domain capable of recognizing this mutated sequence, which would bring along a desired enzymatic activity and restore a proper activity of the locus. Inspiration on how to develop such tools came from the bacterial world. Plant pathogenic bacteria of the *Xanthomonas* genus secrete transcription activator like (TAL) effectors that recognize the promoter sequences of host genes that encode factors that help the bacteria enter the cells [405]. The unique alignment between a repeated amino acid sequence in TAL and bases in the DNA sequence renders TAL amenable for encoding any sequence specificity and targeting TALE to a locus of choice [406]. Similarly, repetitive zinc finger (ZF) domains from the TFIIIA *Xenopus* oocyte extracts [407] can be engineered to recognize a desired sequence in the genome [408,409]. Fusion with *Fok*I nuclease domain produces zinc finger nucleases (ZFN) [410] and TAL-Like Effectors (TALE) [411] and allows to perform highly specific genome modification including gene knockouts in various contexts [412,413,414]. Alternatively, fusion between ZFs and activation domains such as VP64 yields a tool for a specific up-regulation of desired loci [415]. However, despite advances in cloning strategies [411,416], TALEs remain experimentally cumbersome, requiring extensive and often challenging cloning.

**Figure 2:**
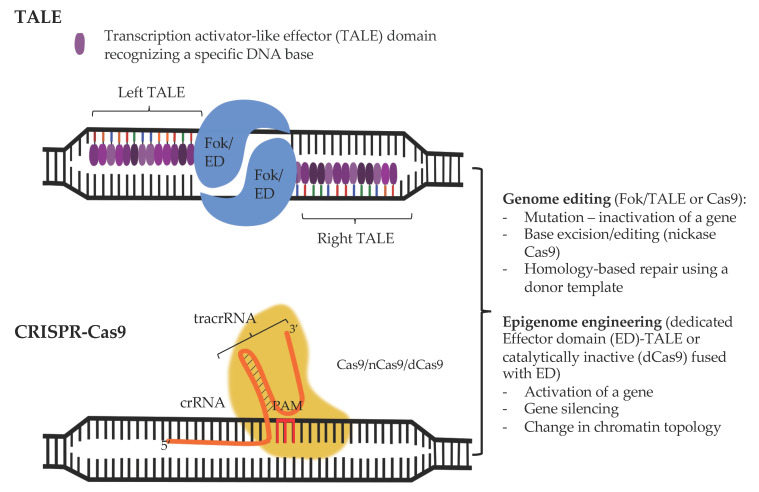
Mechanisms of action of TALE (upper panel) and CRISPR-(d)Cas9 (lower panel). In the TALE system, a designed protein chimera, consisting of transcription activator-like domains (TAL) each recognizing a specific base in the DNA molecule, binds to the target sequence. Fused with a chosen effector domain (ED), TAL effectors (TALEs) can tether a desired enzymatic activity to the target site. This way TALEs can be used to modify the DNA sequence (when endonuclease FokI is the ED) or alter the chromatin landscape. The later can be achieved by fusing TAL with for instance VP16 transactivation domain or the enzymatic domain of p300 HAT allowing to enhance gene expression. Fusion of TAL with KRAB and MeCP2 can induce locus silencing. In CRISPR-Cas9 system, a single guide RNA molecule (sgRNA: guide RNA fused with tracrRNA), harboring a region complementary to the locus of interest, stabilizes the binding of the Cas9 endonuclease and favors the introduction of a double- (Cas9) or a single-strand (nickase-Cas9) DNA break at the recognized site and mutation in the target DNA sequence. When using a catalytically inactive Cas9 (dCas9), this system can also be harnessed to tether an ED to a locus of interest. The dCas9-based epigenome engineering can also be used to modify genome topology and re-introduce promoter-enhancer loops.

More recently the discovery of the prokaryotic acquired immune-like system relying on the Clustered Regularly Interspaced Short Palindromic Repeats (CRISPR) and CRISPR-associated protein (Cas) referred to as CRISPR-Cas machinery allowed the development of a more straightforward and versatile genome editing system (Figure 2). 

### 3.1. CRISPR-Cas-Based Genome Engineering Principles and Considerations 

Prokaryotes safeguard pieces of the genomic DNA (spacers) of their invaders within the CRISPR loci [417,418,419,420]. Transcription of CRISPR genes produces non-coding RNA molecules (crRNA) which are complementary to the genetic material of the intruder (protospacers) [421]. The crRNA, together with the transactivating CRISPR RNA (tracrRNA) [422], allows to specifically target the Cas endonuclease to the invader’s genome [423]. There are several families of Cas proteins, here we will focus on factors from the Class 2 CRISPR–Cas systems such as the Cas9 from Staphylococcus aureus or Cas12a (Cpf1) [424] from, for example, Lachnospiraceae bacterium which are currently most widely used tor (epi)genome modifications. Due to its small size (<1000amino acids), CasX [425] constitutes a great alternative to Cas9 and Cas12a and will probably be extensively developed in the future. 

As we saw above, Cas proteins are tethered to the foreign genome by direct Watson-Crick base pairing between the crRNA and protospacers. The crRNA is 20 or 24 base pair long in Cas9 and Cas12a systems, respectively [422]. Such complementarity is, however, not sufficient to induce nucleolytic reaction and DNA can be cleaved only at sites where the protospacer sequence is flanked downstream by a specific protospacer adjacent motif (PAM) [426,427]. PAMs for distinct Cas proteins differ in composition Cas9 and Cas12a require a G- and T-rich PAMs respectively [428]. PAM is not present at the CRISPR genes, which avoids their destruction and autoimmunity.

Cas9 protein is composed of six domains including REC I, which binds the RNA sequence, REC II, Bridge Helix, PAM Interacting, and HNH and RuvC domains which cleave the single-stranded DNA in a specific manner: HNH attacks the DNA complementary to the crRNA while RuvC cuts the non-target strand [426,429,430], Cas9 produces blunt end double stranded DNA brakes (DSB) [428]. Cas12a lacks an HNH domain and contains the RuvC and a Nuc endonuclease domains, which introduce staggered DNA breaks into the two strands of the target sequence leaving cohesive ends with 4-5 nt. 5′ overhangs [428]. Single molecule approaches, using naked DNA as template, revealed that the Cas9 protein constantly scans the genome; yet only upon the recognition of the PAM sequence does the Cas9 initiate the unwinding of DNA and the hybridization between DNA and the spacer sequence containing RNA [427]. 

The adoption of the CRISPR-Cas system to mammalian cells rendered it possible to engineer the genome sequence by removing or inserting desired fragments of DNA in an efficient manner [431,432,433,434,435,436,437,438,439,440,441,442]. In the Cas9-based system, the procedure relies on a sequence-specific, 20bp-long guide RNA (gRNA) which is covalently modified to include tracrRNA moiety. This construct is sufficient to specifically target the Cas9 endonuclease to the genome and induce double strand breaks [433,434,443]. Cas12a requires solely the gRNA sequence to recognize and cleave DNA [428,440,442]. The number of sites that are targetable by CRISPR-Cas9 system is intimately linked with the the frequency of the PAM and in the human genome the 5′NGG3′ site required for the Cas9 occurs every 8-12 bp [426,427,444].

The genetic damage caused by CRISPR-Cas activates DNA repair mechanism(s) including the non-homologous end joining (NHEJ), microhomology-end joining (MHEJ), as well as homology-directed repair pathway (HDR) that requires an external template to mend the damage [434,445]. The error prone NHEJ pathway may lead to mutational insertions and deletions (indels) of short sequences at the targeted site. Indels can result in gene disruption by for instance frameshift mutation leading to a premature termination of translation and functional disruption of the gene. This property is widely used to silence loci, including in high-throughput genetic screens [446,447,448,449]. However, caution must be taken when designing and interpreting the results as NHEJ can lead to an emergence of gene isoforms with unclear functions [450]. 

Until recently, NHEJ was thought to prevail the homology-assisted mechanisms in resolving DSBs induced by programmable nucleases [451]. However, several reports indicate that MHEJ could in fact constitute the major pathway repairing DSB induced by Cas [452,453]. Moreover, MHEJ would allow us to achieve a remarkable precision in DNA repair [452]. For instance, at loci where frameshift mutations arise as a consequence of microduplications, MHEJ can lead to an efficient removal of the alteration and restoration of gene expression [454]. 

One can tilt the balance of how Cas9-induced DSB are resolved towards homology-based mechanisms including MHEJ. This can be attained by choosing the most optimal cut site at the locus of interest that would lead to MHEJ [452,454]. The most straightforward approach to control the genetic engineering process, however, implicates HDR pathway and is applicable to cells that cycle actively. HDR relies on the external provision of DNA donor sequence with homology to the target site. The HDR pathway mediates the insertion of the desired sequence, provided it is flanked by sequences homologous to the DNA surrounding the cut site. Thus, HDR mediates precise and controllable modification of the genome including a replacement of the mutated nucleotides, addition of an entire domain to an otherwise mutated locus or insertion of a new regulatory sequence in a vicinity of a silenced gene. It allows the inclusion of a transgene into either the site of interest or into the so-called safe-harbor locus which, by mitigating epigenetic silencing, robustly drives gene expression. The induction of the HDR machinery at the CRISPR-Cas-cut sites appears as the most optimal approach: it allows to precisely control the genetic modification and insert a desired sequence at the chosen location. Yet, it requires the cells to pass though the G2/S phase [455] which limits its applicability to NDs. (There are approaches that help increase the efficiency of HDR the reader may consider a recent excellent review on this topic by Yeh and colleagues [445].)

The intricacy of the genome engineering systems and reduced applicability in post-mitotic cells advocates for, where possible, somewhat simpler systems to orchestrate gene expression without the need to modify the DNA sequence. These include programmable nuclease—based approaches that allow to tether a desired enzymatic activity to a precisely defined site in the genome (Figure 2).

### 3.2. From Genome to (Epi)genome Edition

The binding of the Cas9 protein to the genome is stabilized at sites complementary to the gRNA. Like we saw in the case of TALENs and ZFNs, where TALs or ZFs brought the covalently linked *Fok*I nuclease to a specific site in the genome, the DNA specificity of the CRISPR-Cas system can be harnessed to tether a desired activity provided its own endonuclease function is blocked. In *Streptococcus pyogenes* Cas9, this can be achieved by a simultaneous replacement of the aspartic acid at position 10 (RuvC domain) and histidine at position 840 (HNH domain) by alanine, which yields the so called catalytically defective Cas9 (dCas9Sp) [426,456]. In *Staphylococcus aureus* the inactivation of the endonuclease activity of Cas9 (dCas9Sa) requires the mutations of both D10 and aspargine at position 580 (HNH domain). Depending on the fused activity, dCas9 becomes a site-specific modifier of the genome from a single base pair editor to chromatin modification inducer or 3D chromatin structure moderator. As we will see below, it might also be favorable to retain part of Cas9 activity, allowing us to induce a single stranded DNA break (SSB, “nick”) in the instead of DSB; it can be achieved by mutating D10 to A leaving the H840 position unchanged.

#### 3.2.1. dCas9-Orchestrated Base Edition in the Genome

Point mutations are a common cause of genetic disorders and the best approach to correct these alterations would, in principle, be to change the single mutated base pair without the need of larger scale replacement of the DNA sequence as in the HDR. Such modification can be achieved by fusing dCas9 or Cas9 with nick activity (nCas9) with cytosine or adenine deaminases. Cytosine deamination converts it to uracil which can be then converted to thymine when mismatch repair systems (MMR) are favored. The latter can be achieved using nCas9 [457,458,459,460]. Adenine base editors inherit from tRNA adenine deaminase (TadA) of *E. coli* which switches adenine to inosine, which is next converted to guanine in the cell [457]. Base edition constitutes a promising tool for precise base exchange at will, especially since it can successfully be applied to postmitotic cells in vivo [461]. 

#### 3.2.2. Site-Specific Epigenome Rewiring to Enhance Gene Expression 

Highly locus-specific up-regulation of gene expression has been developed over the past three decades. Initially, the approach was to take advantage of potent transcriptional activator domains (ActDs), such as virion protein 16 (VP16; from the herpes simplex virus) or the p65 (component of the NFκB pathway) and target them to the chosen sites in the genome with a factor that is able to specifically recognize DNA sequence such as yeast GAL4 [462] or later ZFs [463,464,465]. Binding of such a construct to its cognate location results in tethering of the transcriptional machinery and gene onset. More recently, ActDs were fused to TALE effectors and dCas9 rendering this tool much easier to use [411,412,466,467]. Multiple recent improvements of the locus-specific activation render this approach more robust, versatile, technically simple, and controllable. 

Loci may appear difficult to modulate using ActD and several strategies allow to alleviate this problem. For instance, the inclusion of ActD arrays as in the VP48, VP64, VP160 or VP192 constructs, built by multiplying the VP16 domain can help increasing the transactivation effect [411,466,468]. In SunTag technology multiple peptides, each of which is recognized by ActD, are fused to dCas9 and altogether enhance gene expression at the locus complementary to the gRNA [469]. Another strategy consists in combining several unique and potent ActDs, as for example in the tripartite activator: VP64-p65-Rta (VPR) [470]. Likewise, an increase in the number of the tethered TALE [467] or dCas9- activators per locus, by for instance inclusion of multiple distinct TALEs or sgRNAs, respectively, and can be used to boost the transcription of the target gene [466]. 

To further increase the versatility of the approach, ActDs may also be replaced with domains displaying enzymatic activities allowing to directly remodel the local chromatin environment at a regulatory stie. Fusion of dCas9 with histone-modifying domains, for instance the HAT domain of p300 [471,472,473] or CBP [474], yields a potent gene expression activator that functions when tethered to both promoters and enhancers. This expands the possibility to address the deficiencies in gene activation due to mutations in regulatory elements including promoters and enhancers [471]. This remarkable tool can be also used to screen for functional regulatory elements in a cell type of choice ([475] for review see [476]). 

Local (epi)genome rewiring may also be achieved by appending histone methyltransferase domains to dCas9. Bringing SET domain of the MLL3/4, which establishes H3K4me1 at enhancers, allows to attract cohesin complex thereby inducing the remodeling of the 3D chromatin arrangement at the locus correlating with the onset of the target gene expression [477]. Furthermore, the fusion of dCas9 with PR/SET Domain 9 that deposits H3K4me3 mark correlating with promoter activity, allows to establish and maintain gene expression [478]. 

Aberrant DNA methylation can lead to erroneous gene silencing with profound molecular consequences. Fusion between site-specific ZFs or dCas9 with either Ten-Eleven Translocation methylcytosine dioxygenase (Tet) or Thymine DNA glycosylase (TDG), enzymes that have been linked to demethylation of methylcytosine, can lead to reactivation of silenced genes [479,480] and disease rescue [481,482,483]. 

**Figure 3:**
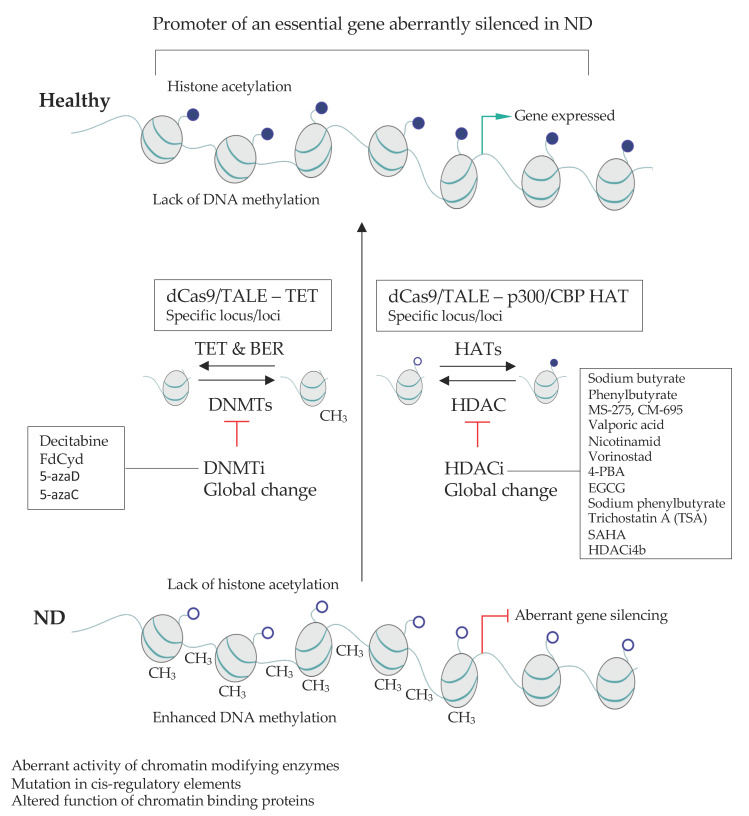
NDs frequently feature altered chromatin landscape including promoter silencing and gene repression. Global or locus-specific approaches can be envisioned to restore proper gene activity. Epidrugs, including histone deacetylase inhibitors (HDACi) or DNA methyltransferase inhibitors (DNMTi), exert a global impact on chromatin and can help reactivate a proportion of aberrantly silenced genes. Designer nuclease-based systems (TALEs and CRISPR-Cas9-based) modify chromatin structure locally and specifically impacting by and large only the chosen loci. Designer nuclease-based systems display minimal off-target effects and potentially extremely low toxicity.

Transcriptional regulation relies on a concert action of TFs, enzymes and proteins that recognize modified chromatin and signal the appropriate conditions the transcriptional apparatus. As we saw above, nonselective HDAC inhibition my exert neuroprotective effects which can be accompanied by memory and learning enhancement. This effect is, in part, mediated by the onset of the expression of BDNF due to the increased histone acetylation arising as a consequence of HDAC inhibition [484,485]. Tethering BRD4, a BET bromodomain containing reader of acetylated histones, to the BDNF promoter further enhances the effect of HDAC inhibition and promotes learning [486]. Thus, the combination of epi-drug treatment with tailored dCas9-mediated epi-targeting might potentially constitute a beneficial clinical avenue (Figure 3). 

#### 3.2.3. Approaches for Locus-Specific Gene Silencing

There are multiple domains that can exert an inhibitory impact on chromatin and thereby gene expression. These include the Krüppel-associated box (KRAB) [487], ERF repressor domain (ERD) [488], mSIN3 interaction domain (SID) [489], friend of GATA1 (FOG1) [490], DNM3A [490], EZH2 and G9A methyltransferases [490,491] along with MeCP2 [492] and HDAC [493]. Echoing designer activators, multiplying repressor domains (RDs) [494] or combining several distinct RDs may help intensify the inhibitory effect on transcription of the target gene [492,495,496]. Mechanistically, repression depends on KAP1/TIF1beta/HP1a pathway in the case of KRAB repressors [497] and additional not yet fully understood mechanisms implicating H3K9me3 and H3K27me3 and DNA methylation [490,493].

Like in the case of site-specific ActDs, the first tailored repressors were recruited to the locus of interest by GCN4 or ZFs [465,487,498]. Later, TALE- [495,499,500] and CRISPR-dCas9-based approaches emerged [490,491,501,502,503]. Due to experimental ease, dCas9 based repressors are currently most widely used in the field. One important difference between ActD and RepD is the stability of the effect. In part, this difference might arise as a consequence of the intrinsic mechanisms that retain DNA methylation during the S-phase of the cell cycle, RD may induce long term effect on gene expression [493] even in cycling cells [498].

#### 3.2.4. Complex Designs to Simultaneously Activate and Repress Distinct Sets of Genes

Transcriptional deregulation in disease is often a complex phenomenon featuring aberrant up- and down-regulation of genes. Thus, simultaneous activation of a subset of loci and inhibition of other targets appears as an attractive and desirable clinical design. It is possible to attain it in a single cell by combining distinct Cas orthologs (dCas9Sp and dCas9Sa for instance) fused to ActD or RepDs [504]. Another approach consists in combining CRISPR-dCas9 system with viral aptamers which recognize specific RNA sequences encoded within the gRNA construct (PP7, MS2, Com or PBS). The aptamers are fused with distinct effector domains: ActDs or RepDs [505,506]. 

#### 3.2.5. Engineering Genome Topology to Orchestrate Gene Expression

One way to orchestrate gene expression is to create a chromatin loop that connects promoter to an enhancer. Artificial loop can be built for instance, via an incorporation of transcription-factor binding sites (TFBS) close to the two sequences that are desired to be in proximity. In Nolis et al. the insertion of TFBS for λ repressor, a prokaryotic TF, close to enhancer and promoter of *INF1* gene, activates its transcription upon expression of the λ repressor [507]. Similar effect was achieved by engineering fusion protein between ZFs recognizing β-globin enhancer and the LIM Domain Binding 1 (LDB1), a factor that interacts with a broad array of TFs that contain LIM domain and mediates interactions between them. Expression of this fusion protein leads to a formation of a loop bridging the β-globin enhancer and promoter leading to a transcriptional up-regulation of β-globin [508].

An artificial loop can be also built using dCas9 heteromonomers or dCas9 monomers fused with a dimerization domain [509]. In this system, heterodimerization between the two fused dCas9 proteins is obtained by adding a chemical compound such as abscisic acid which stabilises an interaction between them. Alternatively, dimerization of dCas9-CIBN and dCas9- CRY2 proteins can be induced by blue light [510].

### 3.3. From Mutation to Precise Target CRISPR-Cas-Mediated In Vivo (Epi)genome Editing to Target NDs

Adeno-associated virus (AAV) delivery-based systems deliver Cas9 into post-mitotic neurons in the brain and has been used to for example study the functional consequences of the removal of DNA-methylation related machinery [511]. This result paves the way to the future possibility to engineer genomes in ND patients. (Epi)genome engineering of essential loci critically related to NDs have been initiated and show a great potential as new therapeutic agents (Table 3). 

For instance, in the Fragile X-syndrome (FXS), FMR1 gene is silenced due to a local expansion of a repeated sequence leading to DNA methylation. Bringing Tet1 to the FMR1 promoter, in post-mitotic neurons derived from FXS patient’s induced pluripotent stem cells, can rescue neuronal functions [527]. Several other essential examples highlight a tremendous potential of (epi)genome rescue tools to address NDs (Table 3). Undoubtably, the strongest arguments in favor of Cas9-mediated treatment approaches are the robustness, specificity, versality and relatively high efficiency of the system Arguments against is the difficulty is the potential for off-target effects and the danger of some yet unknown irreversible consequences of the therapy. The latter being particularly difficult to address, unless sequencing of the whole patient’s genome becomes a routine. 

The delivery of the machinery to the CNS might reveal to be challenging. Lentiviruses (LVs), adenoviruses, adeno-associated viruses (AAVs), as well as non-viral vehicles including extracellular vesicles (EVs), nanoparticles or hybrid formulations have successfully been used as carriers of (epi)genome editing tools in rodents [483,528,529,530]. Viral-based strategies rely on vectors lacking genes essential to viral propagation including rep and cap. Due to that, viral genome remains episomal for a long period of time supporting a continuous gene expression especially in non-dividing cells [530]. LVs are particularly suitable for CRISPR/Cas9 delivery [531], owing to their capacity to carry large DNA inserts including (d)Cas9Sp. Moreover, LVs can transduce a broad range of both dividing and nondividing cells, with minimal cytotoxic and immunogenic response [483]. However, LV are limited in the range of potential target cells (compared to AAVs), tissue penetration and efficiency at doses relevant in vivo [532]. When targeting cells in the CNS, these issues are more challenging. 

A variety of AAVs have also been considered as means of Cas9 delivery to the CNS [533,534,535]. More specifically, AAV2 and AAV9 serotypes appear as the most suitable tools in the context of NDs. As compared to LVs, they possess high brain tissue tropism, desirable infection rate, and lower toxicity, immuno-stimulation, and decreased risk of causing disease in human. Yet, it is unclear how to set the precise level of expression of a transgene using AAVs [536]. In this respect, a transient activation in dividing cells might reveal to be the right approach. 

Despite positive results obtained using AAVs in rodents, there is still a need to enhance the efficiency and robustness of the delivery system. Combination of viral and non-viral carriers could potentially constitute an interesting alternative to the non-hybrid strategies [537,538]. Likewise, more recently, EVs have gained attention in the field as EVs readily pass the BBB and can deliver genome editing tools like Cas9 RNPs and dCas9 RNPs to brain tissue [539] albeit with still relatively low efficiency. The future tools will need to feature larger stability, scalability, BBB and brain tissue permeability, together with the capacity to target chosen cell types in the bran while maintaining safety and optimal (epi)genome-editing efficiency.

#### Essential Considerations When Designing Epigenetic Engineering Strategies 

We have entered an unprecedented era in precision biology, the strategies allowing site-specific modulation of the genome are changing both the fundamental and applied medicine. Yet, several essential points need to be taken into consideration when using ZF/TALE and (d)Cas tools. The efficiency of the reaction is not uniform across the genome and the engineering effect is often markedly affected by the position to which one tethers the effector; this effect impacts both TALE-activators [466,467] as well as dCas9-based factors [466,475]. As we saw above, activators able to recognize stem loops in the gRNA constructs may help diversify the list of genes that could be regulated in a particular cell type simultaneously [475,540] and with lower toxicity [541]. Such systems should also enhance the effector domain activity at challenging sites thereby allowing to bypass the local effects that block ActD or RepD. 

Sustained presence of high concentrations of effector domains may distort cell’s physiology, ActD for instance may hijack transcriptional co-activators thereby impacting on other loci expressed in the cell. To address this limitation, inducible dCas9 systems are being developed for more precise temporal control of gene activation [483] which can be attained with both chemical compounds and light [542]. The core concept of these strategies lies in the capacity to control the heterodimerization of dCas9 and an activator or a repressor protein externally, using tailored extrinsic inducers of interactions between pairs of domains [483,542,543,544,545,546,547]. Alternatively, one can consider quick removal of Cas9 right after induction, KamiCas9 is an interesting approach in which cells are transfected by sgRNA that targets the sequence of interest in addition to a vector encoding sgRNA targeting Cas9 itself. The Cas9 specific sgRNA is expressed under a weak promoter thereby favoring the edition of the target sequence while sustaining the self-inactivating potential [520]. Research in this area is developing extremely rapidly, therefore, we anticipate the development of improved strategies that will be amenable for clinical practice in the nearest future. 

Despite the, in principle, high specificity of ZFs/TALE and (d)Cas, these factors might nonetheless display non-negligeable off-target activity and a potential to introduce modifications at unwanted sites. The off-targets can be predicted using computational tools and directly identified using whole-genome sequencing (WGS) and ChIP-seq experiments allowing to map the regions bound by the dCas9 [548]. Remarkably, WGS-based identification of Cas9 off-target sites revealed an overall low mutation burden (2-2,5% of WGS reads display mutation) [549] and the Cas9 induced off-target indels are produced at a relatively low frequency (below 1-0.1%), primarily within sequences with high homology to the target [436,518,549,550,551]. The design of gRNA can be further optimized using on-line tools which allow to enhance the control over the (epi)genome editing reactions [552,553,554,555].

Altogether, it is essential to experimentally determine which site at a locus of interest is most optimal to attain a desired effect and which approach is best suited to enhance gene expression while keeping cytotoxicity at a minimal level. Likewise, a routine of high throughput sequencing to assess the level of off target edition appears as essential when developing new tools for (epi)genome engineering. 

## 4. Discussion

The remarkable complexity of cell types and cellular interactions in the brain makes it challenging to propose new drugs that could target NDs. Furthermore, very limited regenerative potential of the brain tissue constitutes an additional difficulty in the treatment of NDs featuring neuronal loss such as AD or PD. Finally, the enhanced damming capacity of the BBB needs to be accounted for when designing treatment schemes. Thus, there is a need to develop tailored and specific agents to address NDs at the earliest possible stage.

**Figure 4:**
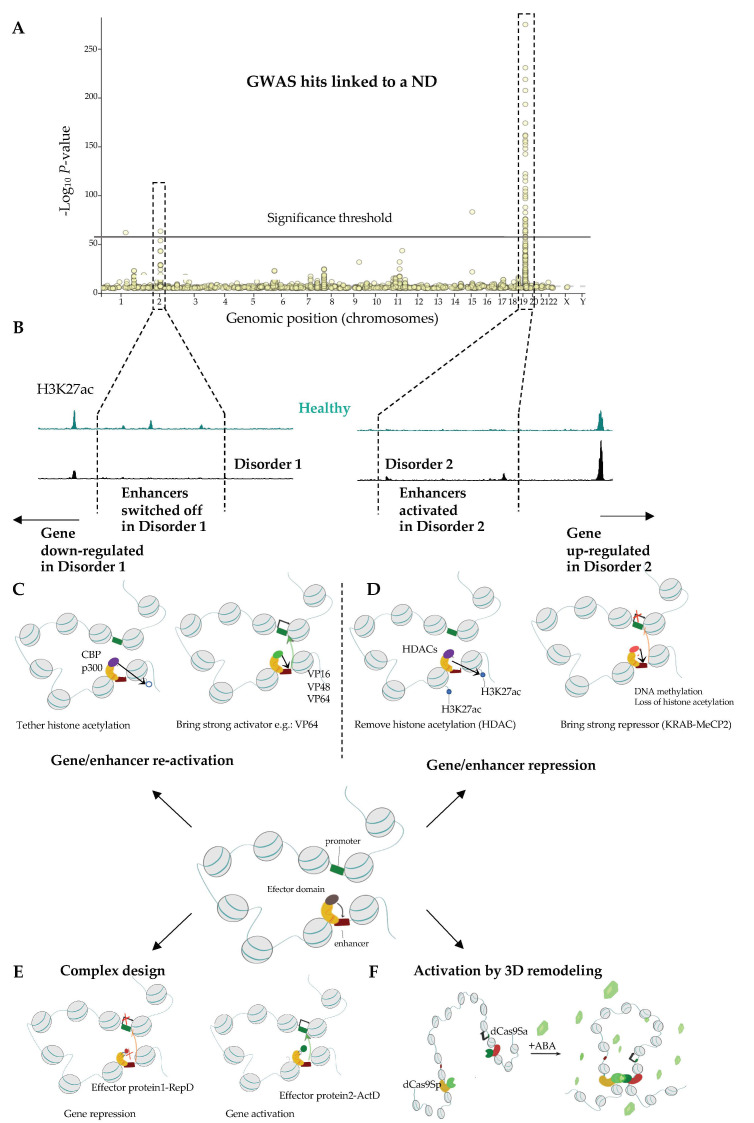
Possible approaches to modulate the activity of the non-coding sequences affected in NDs, including regulatory elements identified in genome-wide association studies (GWAS). (**A**) Manhattan plot displaying an example of GWAS hits linked to a ND. (**B**) GWAS hits may intersect enhancers that can be aberrantly repressed (**left** panel) or activated (**right** panel) in NDs. (**C**) (Epi)genome editing strategies to activate an aberrantly silenced enhancer element in ND. (**D**) Like in (**C**) but displayed are strategies to repress an aberrantly activated enhancer. CRISPR-dCas9 system consists of DNA-binding domain (catalytically deficient Cas9 (dCas9)), and an effector domain which either activates (ActD) or represses (RepD) the activity of a regulatory sequence (here an enhancer). As a consequence, this leads to changes in the expression of the enhancer’s target gene. Gene re-activation is possible via tethering of the dCas9 fused with VP (16, 48, or 64) or the catalytic domain of the p300 HAT to a chosen sequence. Gene repression can be achieved by bringing a dCas9 fused with KRAB, KRAB-MECP2, or HDAC enzymatic domain to the aberrantly activated regulatory sequence. The alternative approach combines the CRISPR-dCas9 system with viral aptamers, recognizing specific RNA sequences encoded within the sgRNA construct (PP7, MS2, Com, or PBS). The aptamers are fused with distinct effector domains: ActD activation domain, RepD- repression domains with activatory or inhibitory impact. (**E**) Complex design allowing to simultaneously repress a set of chosen elements and activate another group of enhancers. (**F**) Gene expression can also be increased due to the induction of the formation of an enhancer-promoter contact. In the CLOuD9 approach, the addition of abscisic acid (ABA) stabilizes the interaction between two complementary dCas9 molecules (Cas9 S. pyogenes and Cas9 s aureus.) tethered to an enhancer and promoter respectively.

Epigenetic drugs that affect the global level of histone modifications in the cell have been used to treat NDs (Table 2, Figure 3). However, this the treatment is unspecific and general and relies on differences in the responses of genes to the global change in epigenetic landscape of the cell. Another approach would be to restore the correct transcriptional pattern of genes mis-regulated in the ND (Figure 4). 

Classical genetic therapies for cancer and other diseases are gaining momentum. CD19-directed chimeric antigen receptor (CAR) T cells are engineered to express a T-cell receptor specifically recognizing CD19 on relapsed or refractory large B cell lymphoma (YESCARTA^®^, NOVARTIS). Other formulations include Zolgensma^®^ which brings a functional copy of the SMN1 gene to treat patients with spinal muscular atrophy. NDs have not yet been targeted with genetic therapies. 

(Epi)genome engineering to restore the correct gene activity or remove a mutated copy of a gene is likely the most attractive future direction in clinical approaches. Can such strategy be envisioned in vivo? CRISPR-Cas9- and TALEN-mediated genome engineering has experimentally been proven feasible in murine model (Table 3) [556] and in the non-human primates [557,558]. The AAV-mediated delivery of the Cas9Sa to mammalian brain appears optimal enough to modulate gene expression and introduce DNA sequence changes. Consequently, there is a number of clinical trials worldwide to address the usefulness of CRISPR-Cas9-mediated genome modification [559]. A study published in June 2021, introduced a lipid nanoparticle-delivered CRISPR-Cas9 genome editing tool to treat individuals affected by transthyretin amyloidosis (ATTR amyloidosis), a life-threatening disease caused by progressive accumulation of misfolded transthyretin (TTR) [560]. This breakthrough finding will likely change the clinical management of monogenic diseases in the future, opening the possibility to edit genomes of patients attained by otherwise untreatable conditions. 

Multitude of TALE/ZF and Cas9-based tools enable the editing of either the DNA sequence or directly gene activity. How can we approach (epi)genome editing? To this end, we need to cartography the regulomes of brain cells and understand how they are distorted in NDs. First step towards this goal is to find and annotate differentially expressed genes and identify their enhancers to promoters in distinct cell types in the CNS. One essential take-home message from the data generated so far is that a single enhancer can regulate multiple genes at a time and that this intricate network of regulatory transactions can be targeted by disease linked GWAS hit [323,342]. Thus, a strategy to restore the correct activity of the GWAS variants might potentially be more fruitful and less toxic to the patient than targeting the signaling pathway affected by the transcriptional mis-regulation of the *cis*-linked genes (Figure 2).

## 5. Conclusions

The understanding of the functional consequences of the mis-regulation of chromatin modifiers will help to determine the most suitable treatment strategies. One thing of course, is to ascertain whether NDs feature a general loss or gain of epigenetic modifications in the genome. In such cases, it seems reasonable to assume that the restoration of the correct global level of chromatin modification represents a potentially advantageous strategy to treat NDs. Yet, one important question is whether such a systemic action is the most suitable strategy or whether a tailored and highly precise approach allowing to restore the correct expression of the chromatin modifier or its essential targets in a particular cellular context will be preferential for the treatment of NDs. The answer will most likely be disease- and patient-dependent.

## Figures and Tables

**Table 1:** Chromatin-related factors implicated in NDs.

Protein	Disease	Phenotype	Reference
DNA Methylases and DNA Methylation-Related Machinery			
MECP2	Rett syndrome	Encephalopathy, intellectual disability (ID), epilepsy	[116,117,118,119]
DNMT1	Hereditary Sensory Neuropathy Type IE—heterozygous mutation		[120]
	autonomic neuropathies with dementia and hearing loss (HSANIE)—heterozygous mutation		[121]
	cerebellar ataxia with deafness and narcolepsy	cerebellar ataxia, narcolepsy/cataplexy, sensorineural deafness, and dementia	[122]
DNMT3a	Tatton-Brown-Rahman syndrome—heterozygous mutation	intellectual disability	[123,124,125]
	Heyn-Sproul-Jackson syndrome—heterozygous mutation	microcephalic dwarfism	[126]
Histone Acetylases			
P300	Rubinstein-Taybi syndrome—heterozygous mutation	microcephaly, mental retardation	[127,128,129]
	Menke-Hennekam syndrome—heterozygous mutation	microcephaly, mental retardation, autistic behavior	[128,130]
CBP	Rubinstein-Taybi syndrome	microcephaly, mental retardation, postnatal growth deficiency	[131,132,133]
	Menke-Hennekam syndrome	microcephaly, Intellectual impairment, autistic behavior	[130,134,135]
MOZ/MYST3/KAT6A	Arboleda-Tham syndrome—heterozygous mutation	microcephaly, impaired intellectual development	[136,137,138,139]
MORF/MYST4/KAT6B	SBBYSS syndrome	mental retardation	[140,141,142,143]
	Genitopatellar syndrome	microcephaly, severe psychomotor retardation	[144,145]
MOF/MYST1/KAT8	Li-Ghorbani-Weisz-Hubshman syndrome—heterozygous mutation	developmental delay, impaired intellectual development	[146]
Histone deacetylases			
HDAC6	dominant X-linked chondrodyspasia	Chondrodysplasia with platyspondyly, distinctive brachydactyly, hydrocephaly, microphthalmia	[147,148]
HDAC8	Cornelia de Lange syndrome	dysmorphic facial features, cleft palate, distal limb defects, growth retardation, developmental delay	[149,150]
SIRT1	anxiety disorder, panic disorder and social phobia	feeling persistent fear, panic attacks, fear of social situations	[151]
Chromatin remodelers			
SWI/SNF complex			
BRM/SMARCA2	Nicolaides-Baraitser syndrome—heterozygous mutation	severe mental retardation, early-onset seizures, short stature, dysmorphic facial features	[152,153]
	Schizophrenia	delusions, disorganized thinking, hallucinations	[154]
	Coffin-Siris syndrome 4	developmental delay, intellectual disability, coarse facial features, feeding difficulties	[155]
BRG1/SMARCA4	Rhabdoid Tumor Predisposition syndrome 2; RTPS1—heterozygous mutation	brain, spinal cord and kidney tumors	[156,157]
BAF47/SMARCB1	Schwannomatosis 1—heterozygous mutation	multiple cutaneous neurilemmomas, spinal schwannomas	[158,159,160,161]
	Multiple meningiomas	meningeal tumor	[158]
	Coffin-Siris syndrome	developmental delay, intellectual disability, coarse facial features, feeding difficulties	[155]
BAF170/SMARCC2	Coffin-Siris syndrome—heterozygous mutation	developmental delay, intellectual disability, coarse facial features, feeding difficulties	[162]
BAF60A/SMARCD1	Coffin-Siris syndrome—heterozygous mutation	developmental delay, intellectual disability, coarse facial features, feeding difficulties	[163]
BAF57/SMARCE1	Meningioma—heterozygous mutation	spinal tumors	[164]
	Coffin-Siris syndrome—heterozygous mutation	developmental delay, intellectual disability, coarse facial features, feeding difficulties	[155,165]
BAF53B/ACTL6B	Developmental and Epileptic Encephalopathy	delayed global development, hypotonia, peripheral spasticity, mainly cerebral atrophy and delayed myelination	[166,167,168,169]
	Intellectual Developmental Disorder With Severe Speech And Ambulation Defects—heterozygous mutation	global developmental delay, impaired intellectual, dysmorphic features: prominent forehead and wide mouth	[169]
BAF250A/ARID1A	Coffin-Siris syndrome—heterozygous mutation	developmental delay, intellectual disability, coarse facial features, feeding difficulties	[155,170]
BAF250B/ARID1B	Coffin-Siris syndrome—heterozygous mutation	developmental delay, intellectual disability, coarse facial features, feeding difficulties	[155,171,172]
BAF200/ARID2	Coffin-Siris syndrome—heterozygous mutation	developmental delay, intellectual disability, coarse facial features, feeding difficulties	[173,174,175]
ACTB	Juvenile-Onset Dystonia—homozygous mutation	eosinophilic, rod-like cytoplasmic inclusions in neocortical and thalamic neurons; abundant eosinophilic spherical structures in the striatum that were strongly actin-and actin depolarizing factor/cofilin-positive	[176,177]
	Baraitser-Winter syndrome—heterozygous mutation	microcephaly, rare lissencephaly or neuronal heterotopia	[178,179]
BAF45D/DPF2	Coffin-Siris syndrome—heterozygous mutation	developmental delay, intellectual disability, coarse facial features, feeding difficulties	[180]
BCL11A	Intellectual developmental disorder with persistence of fetal hemoglobin—heterozygous mutation	delayed psychomotor development, intellectual disability, microcephaly, downslanting palpebral fissures, strabismus, external ear abnormalities, and asymptomatic persistence of fetal hemoglobin (HbF)	[181]
BCL11B	Intellectual Developmental Disorder with Speech Delay, Dysmorphic Facies, and T-Cell Abnormalities—heterozygous mutation	delayed psychomotor development with intellectual disability and speech delay. included autistic features, attention deficit-hyperactivity disorder, anxiety, and other behavioral abnormalities	[182]
BAF180/PBRM1	Autism spectrum disorders	difficulties with social interaction and communication, restricted and repetitive behavior	[183]
ISWI complex			
BRF1	cerebellofaciodental syndrome (CFDS)—homozygous mutation	delayed development, intellectual disability, abnormal facial and dental findings, cerebellar hypoplasia	[184]
BPTF	neurodevelopmental disorder with dysmorphic facies and distal limb anomalies (NEDDFL)—heterozygous mutation	delayed psychomotor development and intellectual disability	[185]
SNF2L/SMARCA1	Schizophrenia syndrome disorders	delusions, disorganized thinking, hallucinations	[186]
	Rett syndrome-like phenotype	Encephalopathy, severe intellectual disability, autistic features, epilepsy	[187]
	Coffin-Siris syndrome-like phenotype	developmental delay, intellectual disability, coarse facial features, feeding difficulties	[166]
BAZ1A/ACF1		Intelectual disability, epilepsy	[188]
CHD protein family			
CHD1	Pilarowski-Bjornsson syndrome—heterozygous mutation	delayed development, intellectual disability, autistic features, speech apraxia, mild dysmorphic features.	[189]
CHD2	childhood-onset epileptic encephalopathy (EEOC)—heterozygous mutation	severe intellectual disability, epilepsy characterized by onset of multiple seizure	[190,191,192,193]
CHD3	Snijders Blok-Campeau syndrome (SNIBCPS)—heterozygous mutation	global developmental delay, impaired intellectual development. Macrocephaly, prominent forehead and hypertelorism, hypotonia, and joint laxity.	[194]
CHD4	Sifrim-Hitz-Weiss syndrome	intellectual developmental disorder	[195,196]
CHD8	Autism spectrum disorders	difficulties with social interaction and communication, restricted and repetitive behavior	[183]
INO80 complex			
YY1	Gabriele-de Vries syndrome—heterozygous mutation	delayed psychomotor development, variable cognitive impairment, behavioral problems, feeding problems	[197]
INO80E		microcephaly, intelectual disability	[198]
SRCAP	Floating-harbor sydrome—heterozygous mutation	short stature, delayed bone age, delayed speech development, facial features	[199]
Cohesin complex			
SMC1	Cornelia de Lange syndrome	intelectual disability, microcephaly	[200,201]
	Developmental and Epileptic Encephalopathy 85 with or without Midline Brain Defects—heterozygous mutation	severe seizures in the first year of life, global developmental delay, impaired intellectual development, poor/absent speech, dysmorphic facial features.	[202,203,204,205,206]
SMC3	Cornelia de Lange syndrome—heterozygous mutation	intelectual disability, microcephaly	[207,208]
RAD21	Cornelia de Lange syndrome—heterozygous mutation	Intelectual disability, microcephaly	[202,209,210,211]
STAG1	autosomal dominant mental retardation-47 (MRD47)—heterozygous mutation	delayed psychomotor development, mild to moderate intellectual disability	[212]
STAG2	Mullegama-Klein-Martinez syndrome—heterozygous mutation	impaired intellectual development, speech delay, hypotonia, microcephaly	[213,214,215]
	X-Linked Holoprosencephaly—heterozygous mutation	incomplete division of the embryonic forebrain	[202]
NIPBL	Cornelia de Lange syndrome	Intelectual disability, microcephaly	[216,217,218]
CTCF	mental retardation-21 (MRD21)	mild intellectual disability, short stature, microcephaly	[219]

**Table 2:** Epidrugs in NDs treatment.

Disease	Epidrug class	Active compound	Model Organism	Remarks	References
Alzeimer’s disease	HDACi	Sodium Butyrate	CK-p25 Tg mice	Improved learning and memory	[371]
			APPPS1-21 mice	Increased histone acetylation, upregulation of neuronal plasticity-related genes, improved associative memory	[372]
		Phenylbutyrate	Tg2576 mice	Improved memory, increased dendritic spines density, Increased histone acetylation	[373]
			N171-82Q mice	Increased H3 and h4 acetylation, mice survival, reduces brain gross and neuronal atrophy	[374]
		MS-275	APPPS1-21 mice	Reduced behavioral impairment, reduced number of amyloid plaques in cortex, enhanced microglia activation in cortex	[375]
		Mercaptoacetamide-based HDACi (W2 and I2)	3xTg AD mice	Decreased alpha amyloid level in vitro, Alpha amyloid synthesis genes downregulation/degradation genes upregulation, decreased Tau protein phosphorylation, improved learning and memory	[376]
		CM-695	Tg2576 Mice	Improved memory, Decreased alpha amyloid level	[377,378,379]
		Valproic acid	APP23 mice	Improved memory, Decreased tau and alpha amyloid	[380]
			Clinical trials		ClinicalTrials.gov Identifier: NCT01729598; NCT00088387
		Nicotinamide	Triple Knock-in mice: presenilin PS1M146V, Amyloid Precursor protein (APPKM670/671NL), Tau (tauP301L)	Prevented cognitive deficits, decreased phosphorylation of tau protein	[381]
			Clinical trials		ClinicalTrials.gov Identifier: NTC00580931; TC03061474
		Vorinostat	Clinical trials		ClinicalTrials.gov Identifier: NCT03056495
		4-PBA	Clinical trials		ClinicalTrials.gov Identifier: NCT03533257
		epigallocatechin-3-gallate (EGCG)	Clinical trials		ClinicalTrials.gov Identifier: NCT00951834
Huntington’s disease	HDACi	TSA/SAHA	*Hdh-Q109* mice	Increased BDNF transport, improved neuron survival	[382,383]
		SAHA	R6/2 HD mouse	improved motor skills	[384]
		Sodium Butyrate	R6/2 HD mice	Improved survival of R6/2 HD mice	[385]
		Sodium butyrate/phenylbutyrate	Human Y39C α-Synuclein Transgenic mouse	Increased expression of DJ1 gene, protection against α-Synuclein Induced Toxicity, reduce α-Synuclein level in mice brain, Prevents Age-related Motor and Cognitive Decline	[386]
		Sodium butyrate, SAHA	D.melanogaster Q48	Decreased neurodegeneration	[387]
		valproic acid	PD patients	Improved myoclonic hyperkinesia	[388]
		HDACi 4b	N171-82Q mice	Decreased Huntingtin aggregation in brain’s cells nuclei, ameliorated cognitive functions	[389]
		MC1568	SHSY5Y Cell line	Enhanced neurite growth of Dopaminergic and Sympathetic Neurons, Increased histone acetylation, Enhanced protection against MPP+ induced neurotoxicity	[390]
	DMNTi	decitabine, FdCyd	In vitro cultured neurons	Reduced cytotoxicity, Increased BDNF expression	[391]
Parkinson’s Disease	HDACi	Sodium phenylbutyrate	Clinical trials		ClinicalTrials.gov Identifier: NCT02046434
		Valproic acid	Rotenone-treated rats	Protection against neurotoxicity	[392]
		phenylbutyrate	SOD1 (G93A) mice	Increased histone acetylation, increased motoneuron survival	[132,393]
Amyotrophic lateral sclerosis	HDACi	trichostatin A	myelin oligodendrocyte glycoprotein peptide-treated WT mice	improved neuronal survival and enhance anti-inflammatory pathway action	[394]
		Vorinostat	myelin oligodendrocyte glycoprotein peptide-treated WT mice	CNS inflammation and demyelination inhibition	[395]
Multiple sclerosis	HDACi	Sodium butyrate	WT rats	Histone 4 acetylation in promotor region of Transthyretin (Ttr), improved performance in FST, TST tests	[396]
		MS-275	WT mice	Increased H3K27 acetylation in accumbens nucleus, decreased expression of stress induced genes	[397]
Depressive disorders	HDACi	MS-275	WT mice	Increased CREB and BDNF expression	[398]
	DNMTi	5-azaD 5-azaC	WT mice	BDNF expression, improved performance in FST test	[396]

Abreviations: HDACi-histone deacetylase inhibitors; DNMTi-DNA methyltransferase (DNMT) inhibitors; 5-azaD-5-aza-2-deoxycytidine; 5-azaC-5-azacytidine.

**Table 3:** (Epi)genome-targeting for treatment of NDs.

Disease	(Epi)genome Modification Strategy and Delivery System	Remarks	References
Parkinson ‘s Disease (PD)	CRISPR-dCas9- DNMT3A targeting α-synuclein Lentiviral vector delivery.	PD patient iPS cell-derived neurons. Silencing of SNCA (α-synuclein) leads to a reduction of disease phenotype downmodulation of ROS * production and an increase in cellular viability.	[512]
	ZF-p65 targeting glial cell line-derived neurotrophic factor (GDNF). AAV2 delivery.	Rat model of PD. Striatal GDNF activation increases neuronal survival.	[513]
Lewy Body Dementia	Combined dCas9-VP64 and sgRNA-MS2-p65 targeting Nrf2 promoter.	In vitro mouse neurons Nrf2 overexpression indued by HDACi and dCas9 ActD helps to clear the toxic deposits of α-synuclein	[514]
Alzheimer’s disease (AD)	ZF fused to G9a, Suvdel76, SKD) or VP64 Dlg4/PSD95.	Mouse. Activation of PSD95 leads to memory rescue in aged and Alzheimer’s disease mice.	[515]
	CRISPR-Cas9 deletion of the mutated allele coding the amyloid precursor protein (APP).	Patient-derived fibroblasts and mouse. Selective silencing of the mutated amyloid precursor protein (APP); 60% reduction in secreted Aβ	[516]
	CRISPR-Cas9-mediated truncation of the C-terminus of the APP AAV9–mediated delivery of CRISPR-Cas9 into mouse hippocampus.	iPS cell-derived neurons, cultured hippocampal neurons, mouse. Removal of the C-terminus of APP results in upregulation of the neuroprotective α-cleavage pathway.	[517]
	CRISPR-Cas9 nanocomplexes Disruption of *Bace1* and *Th* genes in neurons.	5XFAD and *App* knock-in mice mouse. Reduction in Aβ plaque accumulation and Aβ42 secretion. Reduction of Bace1 lead to an increased associative learning and improved spatial working memory and reduced deficit in alternation performance compared with 5XFAD controls. Significant reduction of Aβ42 plaque accumulation and secretion at 8- and 12-weeks post Cas9 injection.	[518]
Huntington‘s Disease	ZFs to block mutant HTT expression AAV2/1 intra-striatal injection.	Cell lines and the R6/2 mouse model. Reduction of diseased clasping behavior, enhanced motor coordination.	[519]
	KAMI-Cas9 targeting HTT gene lentiviral-mediated in vivo delivery.	Neuronal/glial cultures, striatum of mice, and patient-specific iPSC neuronal cells. Decreased HTT aggregation, reduced neuronal dysfunction both in vitro and in vivo.	[520]
	CRISPR-Cas9. rAAV delivery of Cas9.	HD-patient-derived fibroblasts and BacHD mice. Allele-specific removal of mutated HTT gene	[521]
	CRISPR-dCas9MeCP2 downregulation of human HTT AAV-mediated delivery.	Adult brain (striatum) of the mouse HD model (HD140Q-KI). Silencing of the HTT led to reduced neuropathology and increased performance in rotarod, balance beam, and grip strength tests.	[522]
	ZF-KRAB, AAV-mediated delivery.	Patient-derived fibroblasts and neurons, HD mouse models (intra-striatal injection). Improvement in neuronal functions and alleviation of motoneuron symptoms.	[523]
MeCP2 Duplication syndrome	CRISPR-Cas9.AAV-mediated delivery.	Transgenic mouse expressing human MeCP2. Knockout of the human MeCP2. Reversal of the social aversion deficit, yet no impact on the reduced locomotor activity, the heightened anxiety-like behaviors, and the fear generalization phenotype.	[524]
Rett syndrome	CRISPR-Cas9	HR in human iPS cells to correct disease variants.	[525,526]
Fragile X syndrome	CRISPR-dCas9Tet1 demethylation of the *FMR1* gene Lentiviral delivery.	iPS cell model of the Fragile X syndrome Demethylation of the *FMR1* promoter accompanied by a gain of H3K27ac and transcriptional onset in post mitotic neurons derived from the iPS cells	[527]

* Abbreviations: ROS—reactive oxygen species; APP—amyloid precursor protein, HTT—huntingtin, HD—Huntington’s disease.

## Data Availability

Not applicable.
